# Research on reasonable layout parameters of working faces based on the concept of harmonic extraction to reduce the damage of coal seam waiting for upward mining

**DOI:** 10.1038/s41598-023-48822-x

**Published:** 2023-12-08

**Authors:** Chai Jing, Han Zhicheng, Lv Weiming, Zhu Haitao, Hou Shuhong

**Affiliations:** 1 College of Energy Engineering, Xi’an University of Science and Technology, Xi’an, 710054 China; 2 Xi’an University of Science and Technology, Ministry of Education of the Western Mining and Mine Disaster Preventionand Control of Key Laboratory, Xi’an, 710054 China; 3 Zaoquan Colliery, CHN Energy Ningxia Coal Industry Co., Ltd., Yinchuan, 750001 China

**Keywords:** Solid Earth sciences, Engineering

## Abstract

Based on the idea of harmonic extraction, the problem of reducing the damage of the coal seam waiting for upward mining. Using the theoretical analysis method, a schematic diagram of coordinated mining along the dip direction of coal seam is established, and the calculation method of reasonable layout parameters of coal seam working face early mining is given. Based on the upward mining problem of the No.1 coal seam in the fifth panel of Zaoquan Coal Mine, the influence parameters of the No.2 coal seam, No.6 coal seam and No.7 coal seam mining on the No.1 coal seam were determined by similar simulation test. Then, the layout parameters of working face were determined. The research method of numerical calculation was used to evaluate the degree and uniformity of movement and deformation of the No.1 coal seam, combining five indicators: subsidence, horizontal movement, inclined deformation, curvature deformation, and horizontal deformation. The results indicate that when the working face is arranged using the layout parameters provided in this article, it can promote the further subsidence of the No.1 coal seam at the position of the remaining coal pillar. The movement and deformation indicators of the No.1 coal seam all reach the most uniform degree of the geological mining conditions, which can effectively offset the uneven deformation problem of the No.1 coal seam caused by the influence of the remaining coal pillars, making multiple mining operations a favorable condition for upward mining and achieving the goal of reducing the damage of the No.1 coal seam.

## Introduction

Downward mining is a general technical principle for coal seam continuation. When the occurrence conditions of coal seams are special, using the upward mining method can often reduce production costs and the danger during the coal seam mining process^1–3^. The Ningdong mining area is an essential component of the 14 major coal bases that China is focusing on building. Due to the influence of the Jurassic Zhiluo Formation fissure pore interlayer confined aquifer, multiple coal mines adopt upward mining methods to reduce the impact on production. The large number of coal pillars left behind in the early mining of coal seams have led to uneven movement and deformation of the coal seams waiting for upward mining^4,5^, becoming an unfavorable factor for upward mining. It is essential to determine the reasonable layout parameters of coal seam working face early mining, coordinate the mining between coal seams, and achieve the goal of reducing the damage to the coal seams waiting for upward mining. This is of great significance for the rational recovery and utilization of resources and the sustainable development of the mine.

The issue of reducing the damage of coal seams waiting for upward mining shares similarities with the core idea of harmonic extraction to minimize surface damage. Harmonic extraction was initially used to control surface movement and deformation in three-underground mining^6,7^, which involves using multiple adjacent coal mining faces to maintain a particular relationship in time and space to offset uneven surface subsidence and deformation. Research and production practice has shown that coordinated mining can effectively reduce the degree of surface deformation and unevenness^8,9^. Drawing inspiration from the concept of harmonic extraction, this article takes the upward mining problem of the No.1 coal seam in the fifth panel of Zaoquan Coal Mine as the background and comprehensively adopts theoretical analysis, similarity simulation, and numerical calculation research methods to determine the relatively reasonable layout positions of the No.2 coal seam, No.6 and 7 coal seam working faces, which can provide reference for the mining layout design of similar working conditions in mines.

## Method for determining the reasonable layout parameters of the working face

**Figure 1:**
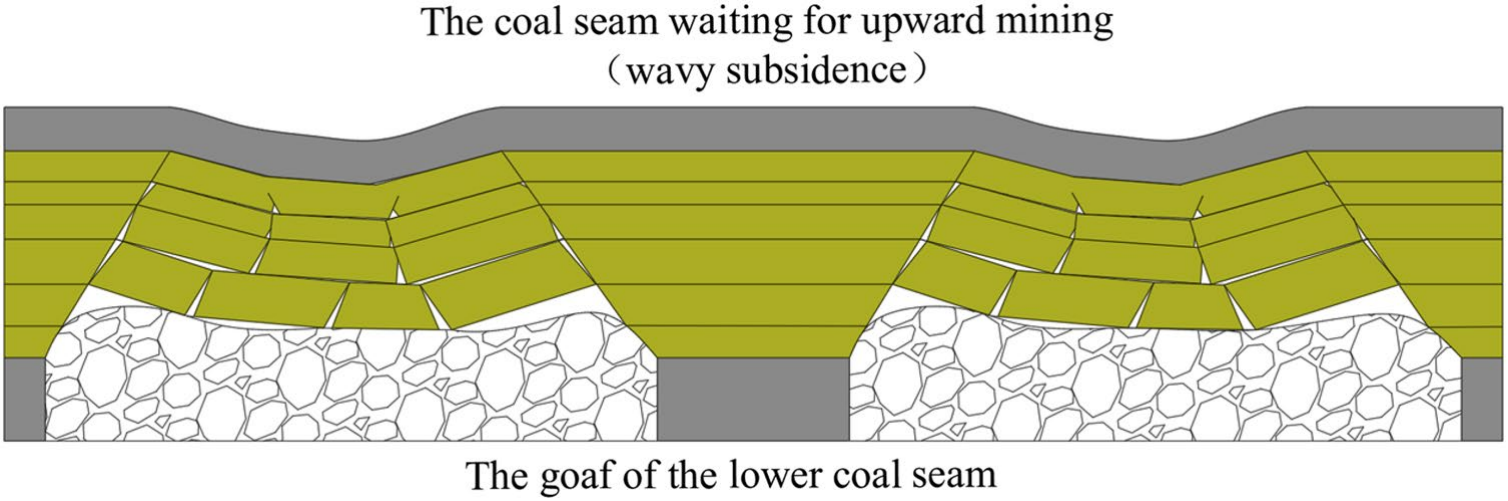
Wavelike uneven subsidence.

Long-term research and production practice have shown that after mining the working face, the movement and destruction space of the overlying rock in the goaf presents a “trapezoidal” shape^10–12^. When using the strike-long wall complete collapse method for coal mining, due to the length of the working face being much smaller than its mining length, a large number of section coal pillars are left in the goaf along the inclined direction of the coal seam after mining^13–15^, due to the influence of the remaining coal pillars, the non-subsidence area, sufficient settlement area, and non-sufficient subsidence area of the overlying rock alternate. The coal seams waiting for upward mining exhibit uneven settlement characteristics in the form of waves in space. Due to the technical requirements of “three smooth and two straights” in the mining face, it has become a disadvantageous factor for upward mining, as shown in Fig. 1.

Drawing inspiration from the concept of harmonic extraction, the essence of reducing the damage to the coal seam waiting for upward mining is to manually adjust the relative position and length between the working faces of the early mining coal seam so that the uneven areas of overlying rock movement and deformation cancel each other out, promote the uniform subsidence of the coal seam, reduce the degree of undulation of the coal seam, and achieve the goal of uniform movement and deformation.

**Figure 2:**
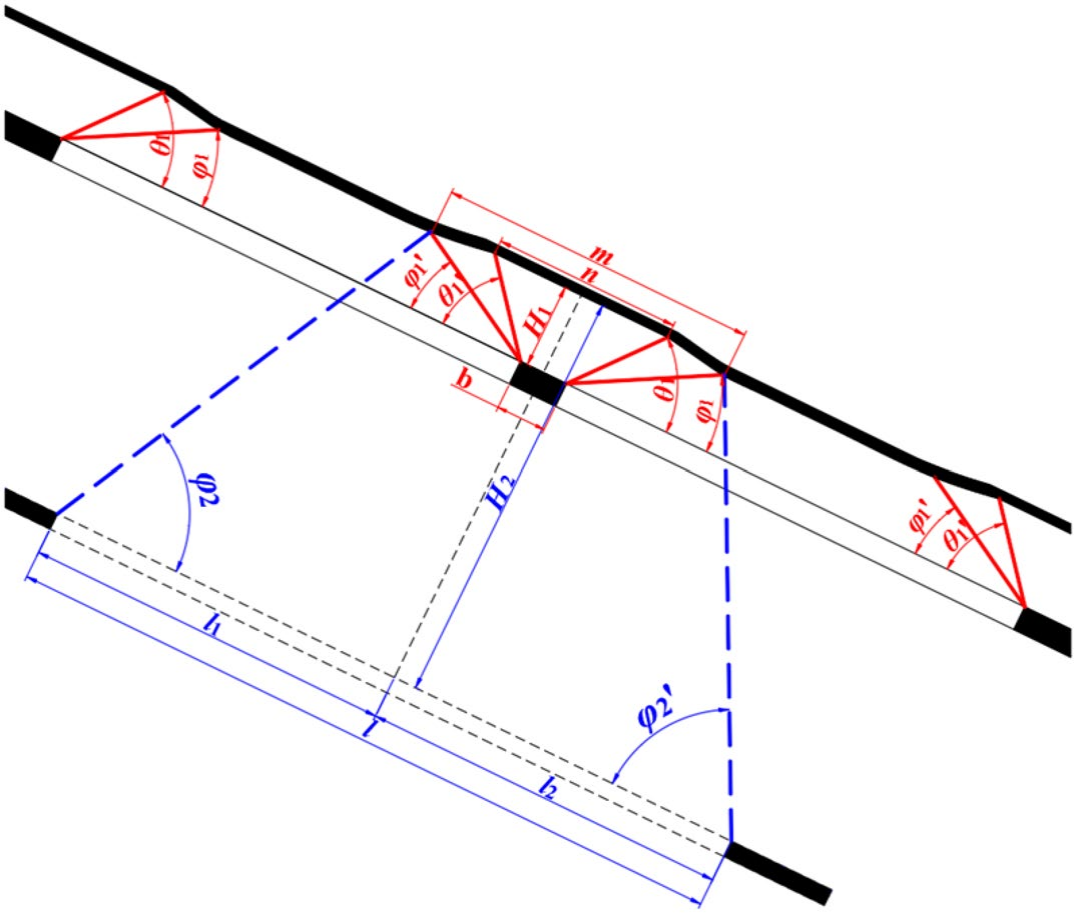
Harmonic extraction schematic diagram.

Compared to mining horizontal or nearly horizontal coal seams, the movement and damage of overlying strata exhibit an asymmetric feature after mining in inclined coal seams. Taking the coal seam waiting for upward mining as a reference, establish a harmonic extraction schematic diagram as shown in Fig. 2, and determine the scope of influence of the working face of the early mining coal seam on the coal seam waiting for upward mining.

After the first layer of coal below the coal seam waiting for upward mining is mined, the coal seam with a range of “n” above the remaining coal pillar is in a non-subsidence state, and the coal seam with a range of “m” is in a non-sufficient subsidence state. According to the geometric relationship shown in 2, its range is calculated using formula 1:

1 $$ \left\{ {\begin{array}{*{20}l} {n = H_{1} /\tan \theta_{1}^{\prime } + H_{1} /\tan \theta_{1} + b} \hfill & {\quad ({\text{Non - subsidence area}})} \hfill \\ {m = H_{1} /\tan \varphi_{1}^{\prime } + H_{1} /\tan \varphi_{1} + b} \hfill & {\quad ({\text{Non - sufficient subsidence area}})} \hfill \\ \end{array} } \right. $$

In the formula, *θ*_1_ and *θ*_1_′ are the subsidence boundary angles of the upper and lower boundaries of the goaf after the first layer of coal mining, °; *φ*_1_ and *φ*_1_′ are the full subsidence angle of the upper and lower boundaries of the goaf after the first layer of coal mining, °; H1 is the interlayer spacing between the coal seam waiting for upward mining and the first coal seam below it, m; B is the width of the remaining coal pillar, m.

After the upper coal seam is mined, the length and position of the lower coal seam working face are controlled to cause the coal seams within the range of [n, m] to subsidence again, thereby weakening the uneven degree of movement and deformation of the coal seam waiting for upward mining. According to the geometric relationship shown in Fig. 2, the distances “*l*_1_” and “*l*_2_” between the upper and lower boundaries of the goaf of the second layer of coal and the centerline of the remaining coal pillars are calculated using formula (2):

2 $$ \left\{ {\begin{array}{*{20}l} {{{H_{2} } \mathord{\left/ {\vphantom {{H_{2} } {\tan \varphi_{2} }}} \right. \kern-0pt} {\tan \varphi_{2} }} + {{H_{1} } \mathord{\left/ {\vphantom {{H_{1} } {\tan \theta_{1}^{\prime } }}} \right. \kern-0pt} {\tan \theta_{1}^{\prime } }} + {b \mathord{\left/ {\vphantom {b 2}} \right. \kern-0pt} 2} \le l_{1} \le {{H_{2} } \mathord{\left/ {\vphantom {{H_{2} } {\tan \varphi_{2} }}} \right. \kern-0pt} {\tan \varphi_{2} }} + {{H_{1} } \mathord{\left/ {\vphantom {{H_{1} } {\tan \varphi_{1}^{\prime } }}} \right. \kern-0pt} {\tan \varphi_{1}^{\prime } }} + {b \mathord{\left/ {\vphantom {b 2}} \right. \kern-0pt} 2}} \hfill \\ {{{H_{2} } \mathord{\left/ {\vphantom {{H_{2} } {\tan \varphi_{2}^{\prime } }}} \right. \kern-0pt} {\tan \varphi_{2}^{\prime } }} + {{H_{1} } \mathord{\left/ {\vphantom {{H_{1} } {\tan \theta_{1} }}} \right. \kern-0pt} {\tan \theta_{1} }} + {b \mathord{\left/ {\vphantom {b 2}} \right. \kern-0pt} 2} \le l_{2} \le {{H_{2} } \mathord{\left/ {\vphantom {{H_{2} } {\tan \varphi_{2}^{\prime } }}} \right. \kern-0pt} {\tan \varphi_{2}^{\prime } }} + {{H_{1} } \mathord{\left/ {\vphantom {{H_{1} } {\tan \varphi_{1} }}} \right. \kern-0pt} {\tan \varphi_{1} }} + {b \mathord{\left/ {\vphantom {b 2}} \right. \kern-0pt} 2}} \hfill \\ \end{array} } \right. $$

After determining the length of *l*_1_ and *l*_2_, the sum of them is a reasonable range for the length of the second layer of the coal working face. When mining multiple coal seams in the early stage, the above calculation method can also be used to determine the layout parameters of the working face. The key lies in adopting effective measurement or experimental methods to determine the impact parameters of early mining coal seams on the coal seam waiting for upward mining.

## Example validations

### Engineering background

The fifth panel of Zaoquan Coal Mine has a length of 4.8 m, a width of 2.0 m, and an area of 9.6 km^2^. The strata in the mining area are anticlinal structures with an average dip angle of 26°–28°. The immediate roof of the No.1 coal seam in the fifth panel is the Jurassic Zhiluo Formation confined aquifer, with an average thickness of 51 m and an aquifer thickness of 23.0–67.1 m. It has good water abundance and is expected to have a water reserve of approximately 1.15 million m^3^. At the same time, the No.1 coal seam is also affected by the wind oxidation zone, with strong water storage capacity on the roof and a water static reserve of approximately 126,000 m^3^ in the wind oxidation zone. The total amount of water in the aquifer and oxidation zone is 1.276 million m^3^, significantly impacting the roadway excavation and working face extraction of the No.1 coal seam. In order to reduce the impact of water in the roof of the No.1 coal seam on production, the sequence of coal seam continuation is adjusted to No.2 → 6 → 7 → 1 coal seam, and the water flowing fractured formed by early mining of the lower part of the No.1 coal seam are used to drain the roof water.

The No.1 coal seam has an average thickness of 2.2 m, the No.2 coal seam has an average thickness of 7.4 m, the No.6 coal seam has an average thickness of 2.4 m, and the No.7 coal seam has an average thickness of 2.2 m. The No.1 coal seam is approximately 25.4 m away from the No.2 coal seam, 125.4 m away from the No.6 coal seam, and 131.4 m away from the No.7 coal seam. The distance between the No.6 coal seam and the No.7 coal seam is about 3.5 m, and the working face is arranged by simultaneously mining the upper and lower coal seams. The stratigraphic information is listed in Table 1.

**Table 1:** Stratigraphic information.

Number	Stratigraphic units and symbols	Rock layer name	Accumulated true thickness of rock layers (m)	True thickness of rock layer (m)
1	Quaternary (Q_4_)	Sandy soil	12.70	12.70
2			21.04	8.34
3	Middle Jurassic Zhiluo formation (J_2_z)	Sandy mudstone	26.14	5.10
4		Fine-sandstone	30.42	4.28
5		Sandy mudstone	34.50	4.08
6		Coarse-sandstones	101.56	67.06
**7**	Middle Jurassic Yan'an formation (J_2_y)	**No.1 coal seam**	**104.06**	**2.50**
8		Fine-sandstone	108.23	4.17
9		Coal seam	108.32	0.09
10		Fine-sandstone	110.36	2.04
11		Siltstone	112.77	2.41
12		Medium-sandstone	114.35	1.58
13		Fine-sandstone	116.53	2.18
14		Medium-sandstone	120.14	3.61
15		Siltstone	121.88	1.74
16		Fine-sandstone	125.10	3.22
17		Coarse-sandstones	125.92	0.82
18		Fine-sandstone	128.39	2.47
19		Mudstone	129.49	1.10
20		**No.2 coal seam**	137.62	8.13
21		Siltstone	144.06	6.44
22		Coarse-sandstones	174.55	30.49
23–1		Siltstone	177.29	2.74
23–2		Coal seam	177.52	0.23
23–3		Siltstone	182.18	4.66
24		Fine-sandstone	184.19	2.01
25		Sandy mudstone	184.66	0.47
26		Coal seam	186.03	1.37
27		Fine-sandstone	188.28	2.25
28		Siltstone	189.06	0.78
29		Coal seam	189.24	0.18
30		Siltstone	189.77	0.53
31		Fine-sandstone	196.19	6.42
32		Siltstone	203.17	6.98
33		Sandy mudstone	204.27	1.10
34		Coal seam	204.45	0.18
35		Fine-sandstone	205.61	1.16
36		Siltstone	209.90	4.29
37		Fine-sandstone	211.09	1.19
38–40		Coal seam	212.64	1.55
41		Fine-sandstone	219.49	6.85
42		Siltstone	220.77	1.28
43–45		Fine-sandstone	224.47	3.70
46–1		Siltstone	229.45	1.42
46–2		Coal seam		0.23
46–3		Coal seam		3.33
**47**		**No.6 coal seam**	**232.01**	**2.56**
48		Fine-sandstone	235.48	3.47
**49**		**No.7 coal seam**	**238.22**	**2.74**

Minable coal seam are in [bold].

### Similar simulation experiments

The movement of overlying rocks above the stope after coal extraction is a typical black box problem. A physical similarity simulation experiment is a model made in the laboratory based on the principle of similarity, which is similar to the prototype. With the help of testing instruments, the internal force parameters and their distribution patterns of the model are observed. The results of research on the model are used to infer possible mechanical phenomena and the distribution patterns of rock pressure in the prototype, thereby solving practical problems in rock engineering production^16–18^. It has been widely applied in the study of overlying rock movement and mine pressure and control in coal mining.

In order to determine the influencing parameters of the movement and deformation of the No.1 coal seam after mining the No.2 coal seam, No.6 and 7 coal seam in the fifth panel of Zaoquan Coal Mine. According to the geological information of the fifth mining area, using sand as the aggregate CaSO_4_ and CaCO_3_ as the cementitious material, a physical similarity model of a plane is constructed. The parameters of the similarity model and material proportions are shown in Tables 2 and 3, respectively.

**Table 2:** Parameters of physical similarity model.

Dip angle	Size (length × wide × High)	Geometric similarity ratio	Stress similarity ratio	Time similarity ratio
26°	2000 mm × 200 mm × 1500 mm	1:150	1:240	1:12.24

**Table 3:** Proportion of similar material masses.

Number	Rock layer name	Sand:CEMENTITIOUS material	CaSO_4_:CaCO_3_	Notes (ratio number)
1	Sandy soil	9:1	1:4	928
2	Mudstone	8:1	2:3	846
3	Sandy mudstone	8:1	3:7	837
4	Siltstone	7:1	3:7	737
5	Fine-sandstone	7:1	1:1	755
6	Medium-sandstone	7:1	2:3	746
7	Coarse-sandstones	7:1	1:4	728
8	Coal seam	20:1:5:20		Sand:CaSO_4_:CaCO_3_:fine-coal

**Figure 3:**
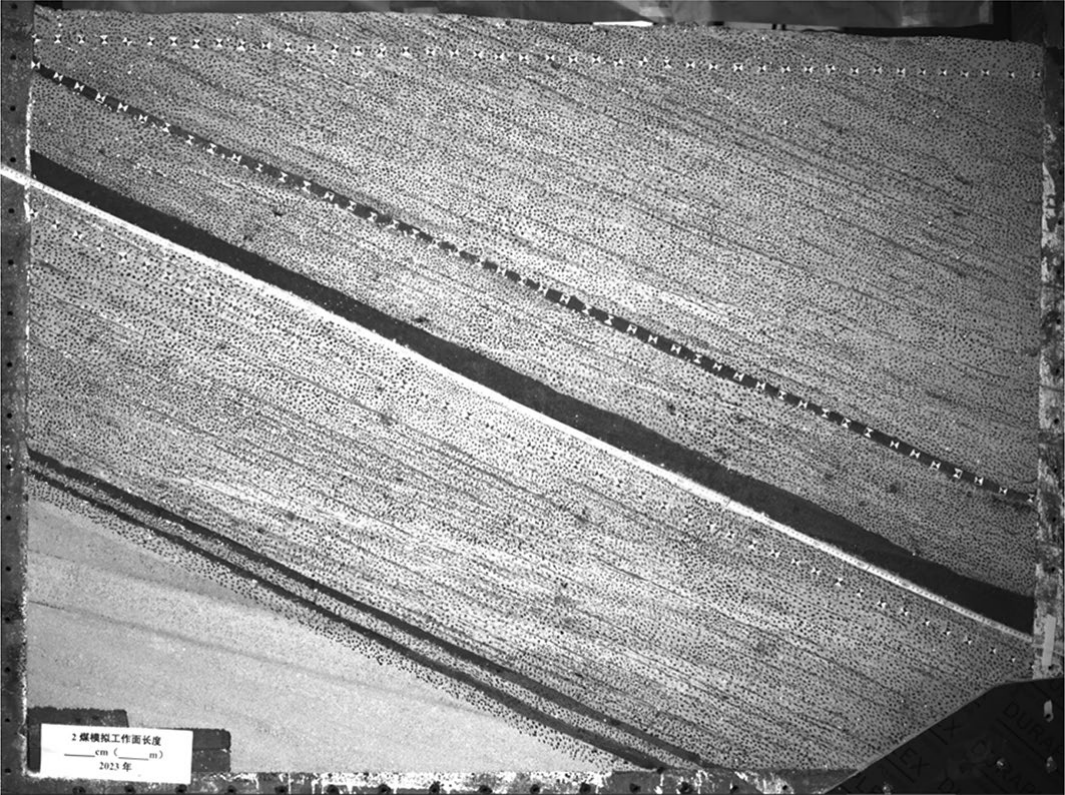
Physical similarity model.

The width of the boundary coal pillar in the No. 2 coal seam is 300 mm, and the maximum simulated mining length is 1600 mm, corresponding to the actual working face length of 240 m. The width of the boundary coal pillar in the No. 6 and 7 coal seams is 100 mm, and the maximum simulated mining length is 1200 mm, corresponding to the actual working face length of 180 m. When simulating coal seam mining, each excavation distance is 100 mm. After each excavation, wait for 20 min to ensure sufficient movement of the overlying rock layer. In order to accurately obtain the movement parameters of the overlying rock and No.1 coal seam after mining No.2, 6 and 7 coal seams. Spray speckles are on the surface of similar models, and measurement points are arranged along the No.1 coal seam at an interval of 50 mm, as shown in Fig. 3. Use a total station and camera to monitor the movement of measurement points and the entire model field.

### Movement and deformation characteristics of overlying rocks and No.1 coal seam

**Figure 4:**
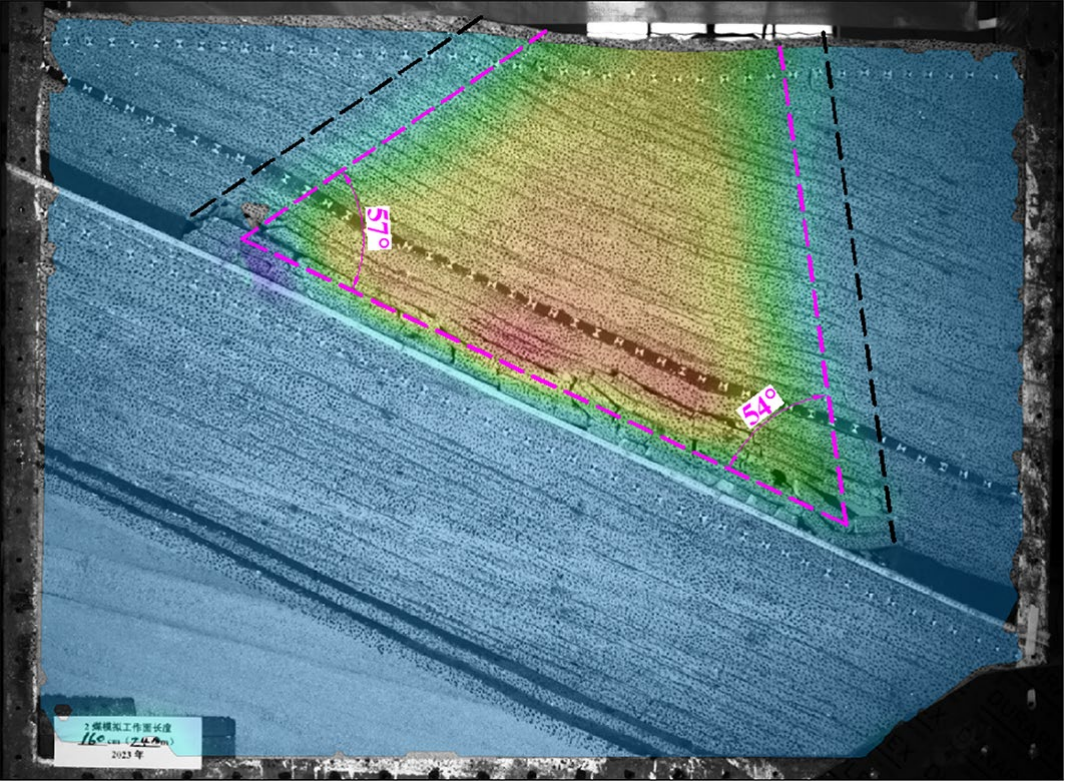
Simulates the movement range of overlying rock after mining of the No.2 coal seam.

After simulating the mining of the No.2 coal seam, the movement range of the overlying rock shows a “trapezoidal” shape, and the caving angle of the overlying rock at the upper boundary of the goaf is about 57°. In comparison, the lower boundary is about 54°. The No.1 coal seam and its underlying rock layers form a hinged structure on both sides of the remaining coal pillar, and there is a large mining space below it. Under the concentrated stress of mining, step sinking may occur, which is a potential hazard source for upward mining of the No.1 coal seam. Compared to the compacted area in the middle of the goaf, the coal seam undulates significantly due to residual coal pillars and articulated structures on both sides, as shown in Fig. 4.

**Figure 5:**
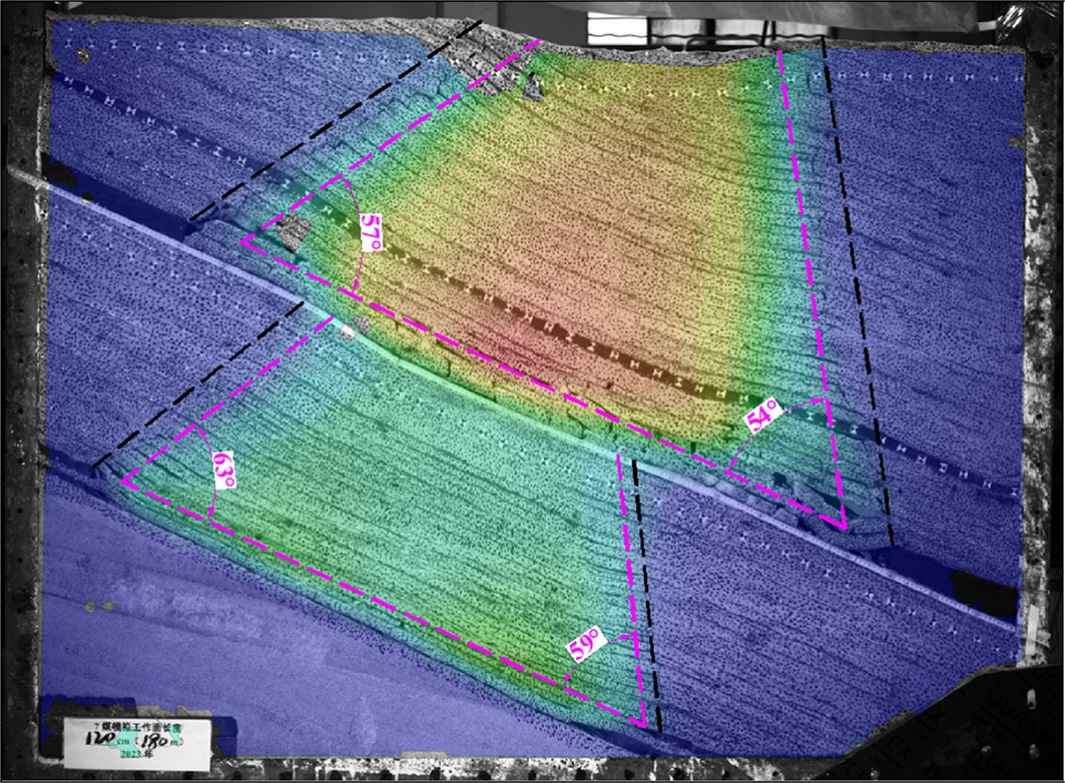
Simulates the movement range of overlying rock after mining of No.6 and 7 coal seams.

After simulating the mining of No.6 and 7 coal seams, the movement range of the rock layers between the coal seams also presents a “trapezoidal” shape. The rock layers' caving angle at the goaf's upper boundary is about 63°, and the lower boundary is about 59°. The No.1 coal seam maintains a relatively complete layered shape as a whole, but due to the caving angle of the overlying rock and the presence of coal pillars, the overall subsidence of the overlying rock on a large scale cannot be guaranteed after the mining of the No.6 and 7 coal seam, resulting in significant fluctuations in the No.1 coal seam at the mining site boundary. At the same time, the rotation of the articulated rock beam below the No.1 coal seam at the boundary of the mining site increases, and the mining cracks further expand, which is a disadvantageous factor for the upward mining of the No.1 coal seam, as shown in Fig. 5.

In order to quantify the degree of movement and deformation of the No.1 coal seam in the model experiment. Calculate the subsidence, inclination, curvature, horizontal movement and horizontal deformation curves of the No.1 coal seam using formulas (3)–(7).

3 $$ w_{n} = H_{n - 0}^{{}} - H_{n - m} $$

In the formula, *H*_*n*−0_ and *H*_*n*−*m*_ are the elevations of measurement point n during the first and the m-th observations, respectively.

4 $$ i_{n\sim n + 1} = \frac{{w_{n + 1} - w_{n} }}{{l_{n\sim n + 1} }} = \frac{{{\Delta }w_{n\sim n + 1} }}{{l_{n\sim n + 1} }} $$

In the formula, *l*_*n*~*n*+1_ is the horizontal distance from measurement point n to measurement point n + 1.

5 $$ k_{n - 1\sim n\sim n + 1} = \frac{{i_{n\sim n + 1} - i_{n - 1\sim n} }}{{\frac{1}{2}\left( {l_{n - 1\sim n} + l_{n\sim n + 1} } \right)}} = \frac{{2{\Delta }i_{n - 1\sim n\sim n + 1} }}{{l_{n - 1\sim n} + l_{n\sim n + 1} }} $$

6 $$ u_{n} = L_{n - m} - L_{n - 0} $$

In the formula, *L*_*n*−*m*_ and *L*_*n*−0_ are the horizontal positions of measurement point n at the m-th and first observation, respectively.

7 $$ \varepsilon_{n\sim n + 1} = \frac{{u_{n + 1} - u_{n} }}{{l_{n\sim n + 1} }} = \frac{{{\Delta }u_{n\sim n + 1} }}{{l_{n\sim n + 1} }} $$

**Figure 6:**
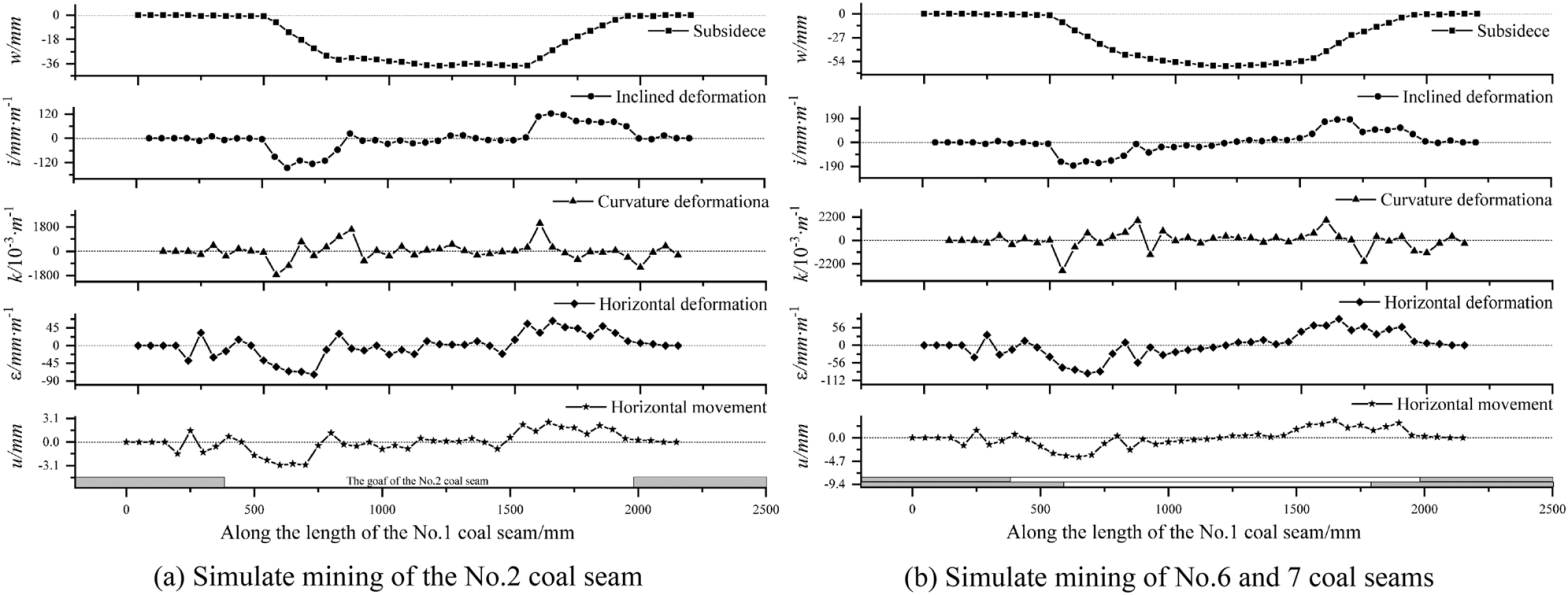
Movement and deformation curve of the No.1 coal seam.

Obtain the movement and deformation curves of the No.1 coal seam after mining No.2, 6, and 7 coal seams, as shown in Fig. 6. After the mining of the No.2 coal seam, the maximum subsidence of the No.1 coal seam is 37.6 mm, the inclination deformation is 145.9 mm m^−1^, and the curvature deformation is 2071.1 × 10^−3^ m^−1^, the horizontal deformation is 73.6 mm m^−1^, horizontal movement 3.0 mm. The subsidence curve of the No.1 coal seam shows a flat bottom “bowl” shape, reaching a sufficient subsidence state. Based on its relative position with the goaf of the No.2 coal seam, the subsidence boundary angles (*θ*_1_, *θ*_1_′) of the upper and lower boundaries of the goaf are determined to be about 55° and 79°, respectively. The sufficient subsidence angles (*φ*_1_, *φ*_1_′) are about 22° and 21°, respectively.

**Figure 7:**
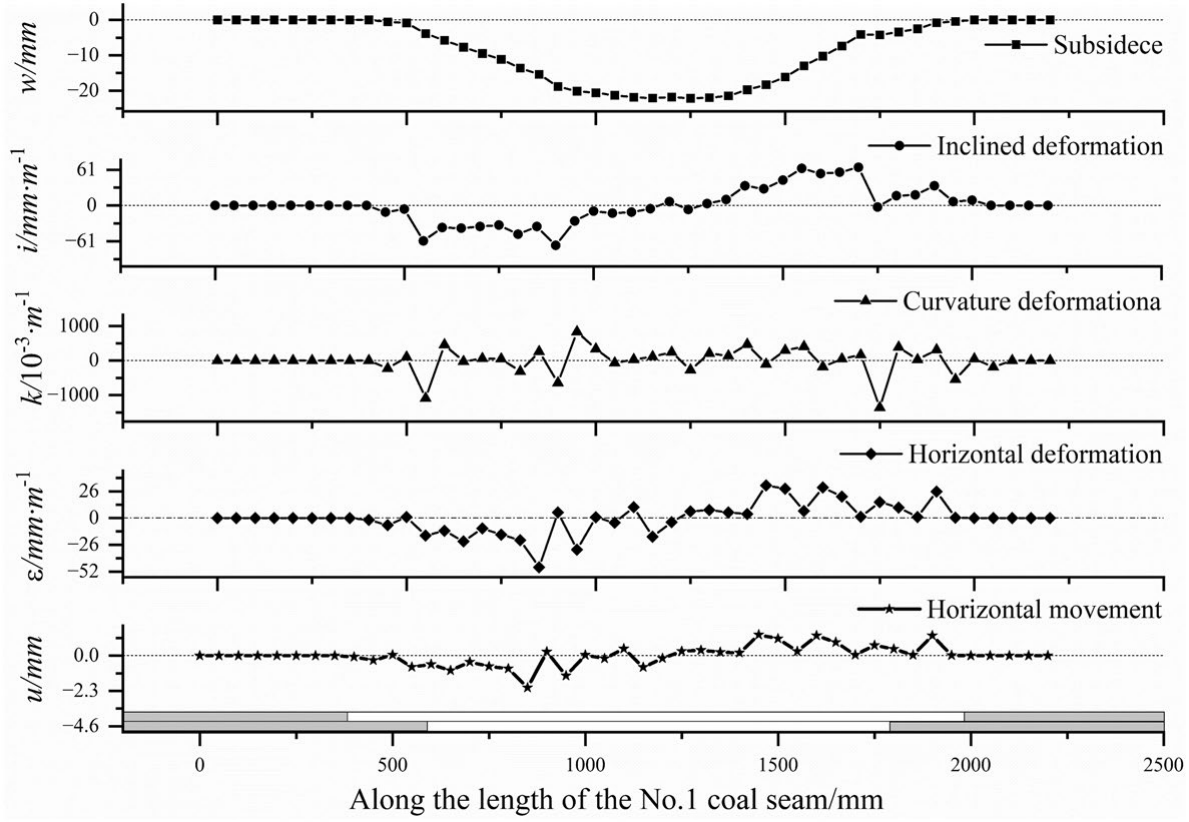
Incremental curve of movement and deformation of No.6 and 7 coal seams.

Figure 7 shows the incremental curve of the movement and deformation of the No.1 coal seam after the completion of the mining of the No.2 coal seam, No.6 and 7 coal seam compared to the completion of the mining of the No.2 coal seam. The maximum movement deformation values of the No.1 coal seam increased to 59.4 mm, 183.3 mm m^−1^, 2838.0 × 10^−3^ m^−1^, 89.4 mm m^−1^, 3.8 mm, respectively, with an increase of 57.9%, 25.6%, 37.0%, 21.5%, and 26.5%. The subsidence increment curve also presents flat bottomed "bowl" shape, with sufficient subsidence angles (*φ*_2_, *φ*_2_′) of about 65° and 66° at the upper and lower boundaries of the goaf of the No.6 and 7 coal seam, respectively.

### Working face layout parameters

**Figure 8:**
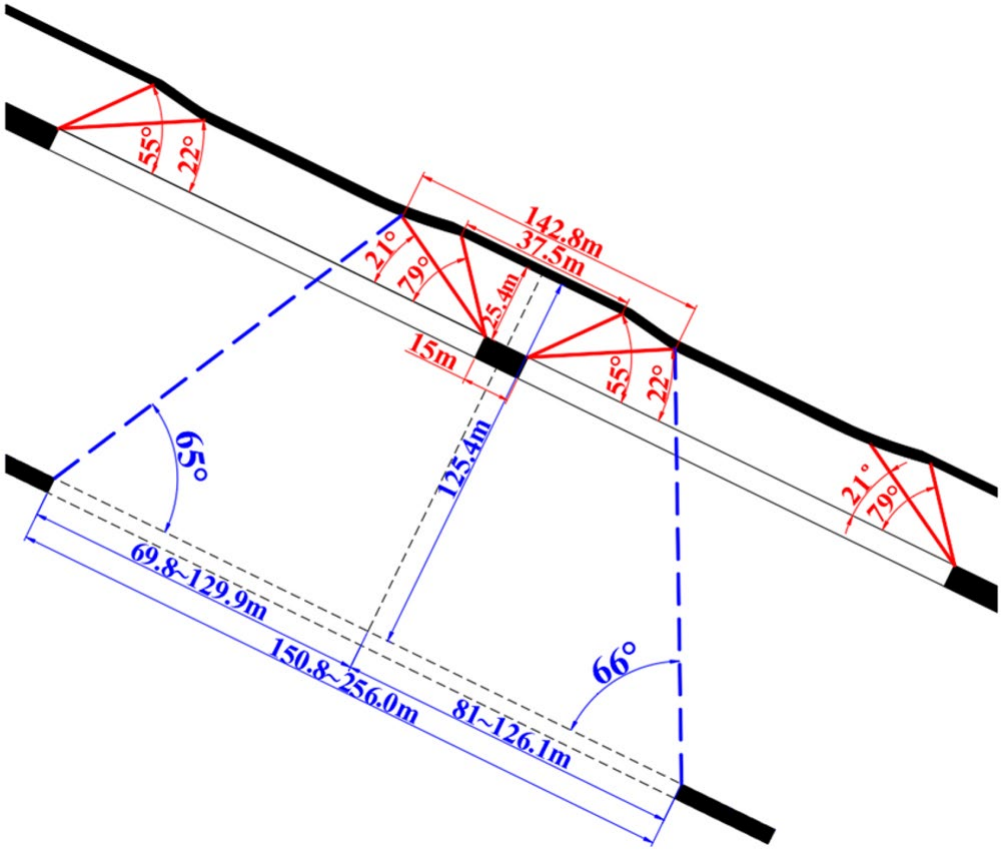
Schematic diagram of working face layout parameters.

According to the existing design of the mine, the working face length of the No.2 coal seam is 240 m, and the section coal pillar width (b) is 15 m. By incorporating relevant parameters into formulas (1) and (2), the non-subsidence range (n) of the No.1 coal seam is 37.5 m. The non-sufficient subsidence range (m) is 142.8 m after the two adjacent working faces of the No.2 coal seam are mined. The distance (*l*_1_) between the upper boundary of the No.6 and 7 coal seams working face and the centerline of the remaining coal pillar in the No.2 coal seam should be between [69.8 m, 129.9 m], the lower boundary (*l*_2_) should be between [81.0 m, 126.1 m]. The length of the working face (*l*_1_ + *l*_2_) should be between [150.8 m, 256.0 m], as shown in Fig. 8.

## Numerical simulation experiment verification

Due to limitations in the size of similar simulation experiments, it is impossible to simulate the movement of overlying strata after large-scale coal seam mining. Using the UDEC numerical calculation software^19–21^, six comparative experiments were established to verify the rationality of the working face layout parameters and calculation methods of the No.6 and 7 coal seams obtained above, as listed in Table 4.

**Table 4:** Experimental scheme.

Experiment number	Working face length/m	The distance between the boundary of the working face and the center of the remaining coal pillar/m		Notes
		Upper boundary	Lower boundary	
I	240	–	–	Mining only the No.2 coal seam
II	240	162	78	The upper boundary is not within the interval
III	240	122	118	Within the interval
IV	240	42	198	The lower boundary is not within the interval
V	100	50	50	The length of the working face is less than the length of the interval
VI	300	150	150	The length of the working face is greater than the length of the interval

As a natural material, rock has characteristics such as discontinuity, heterogeneity, and anisotropy^22,23^. The distribution of primary and secondary joints in rock masses also exhibits randomness^24^. The discrete method is an essential factor that affects the accuracy of discrete numerical calculation methods^25^. In order to reduce the subjectivity and dependency in the discretization process of numerical calculation models and improve the accuracy of numerical calculations, the VORONOI method was used to discretize the model as a whole, followed by secondary cutting along the layered interface of the rock layer to simulate the layered characteristics of sedimentary rocks.

The M–C strength model^26–28^ was selected as the constitutive model for joints and blocks. The M–C model represents the yield limit with linear characteristics, and at the macro level, it exhibits characteristics such as rock elastic stiffness, friction, cohesion, tensile strength, and shear expansion. This model characterizes the failure of strength by losing cohesive and tensile strength after reaching the strength limit. The mechanical parameters of the overlying rock are shown in Table 5.

**Table 5:** Rock mechanical parameters.

Rock layer name	Elastic modulus/MPa	Poisson's ratio	Density/(kg m^−3^)	Cohesion/MPa	Friction angle/°	Tensile strength/MPa
Coal seam	2333	0.32	1357	2.23	27	1.31
Siltstone	20,165	0.26	2438	21.71	35	12.77
Fine-Sandstone	14,200	0.18	2610	21.44	32	12.61
Medium-Sandstone	11,795	0.29	2137	24.16	35	14.21
Coarse-Sandstones	12,300	0.20	2620	20.33	38	11.96
Mudstone	13,900	0.19	2610	21.32	32	12.54
Sandy soil	0.25	0.35	20.00	0.03	18	0.02

**Figure 9:**
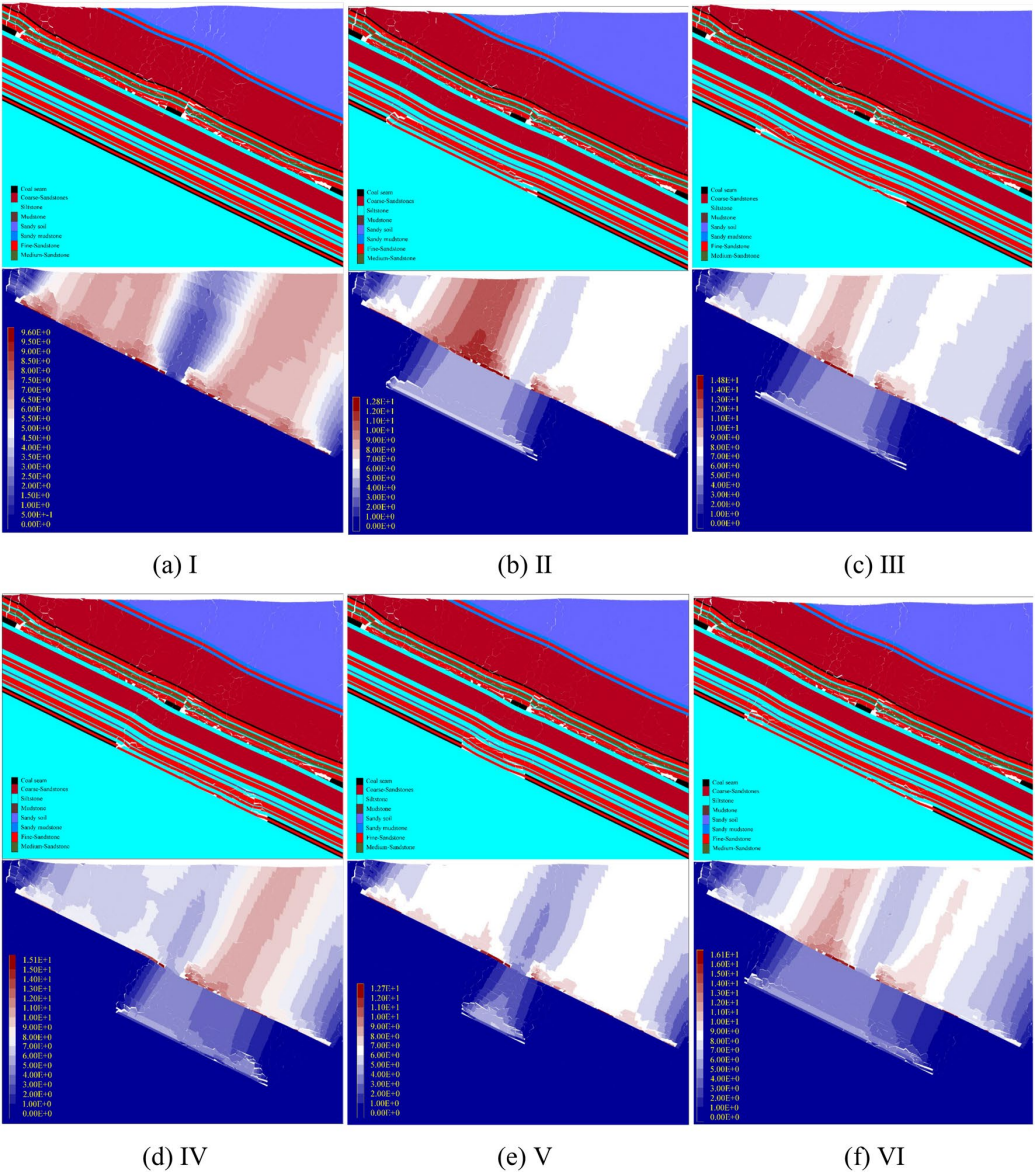
Failure and movement of overburden rock.

Figure 9 shows the failure and movement cloud maps of the overlying rocks in 6 sets of experiments. After the two adjacent working faces of the No.2 coal seam were mined, the overall layer shape of the No.1 coal seam remained relatively intact, with good continuity and local dislocations. The subsidence pattern of the No.1 coal seam within a range of approximately 146 m above the remaining coal pillar is convex and in a non-sufficient subsidence state, which is close to the calculated range. The results of numerical simulation experiments are reliable.

### Movement and deformation of the No.1 coal seam

**Figure 10:**
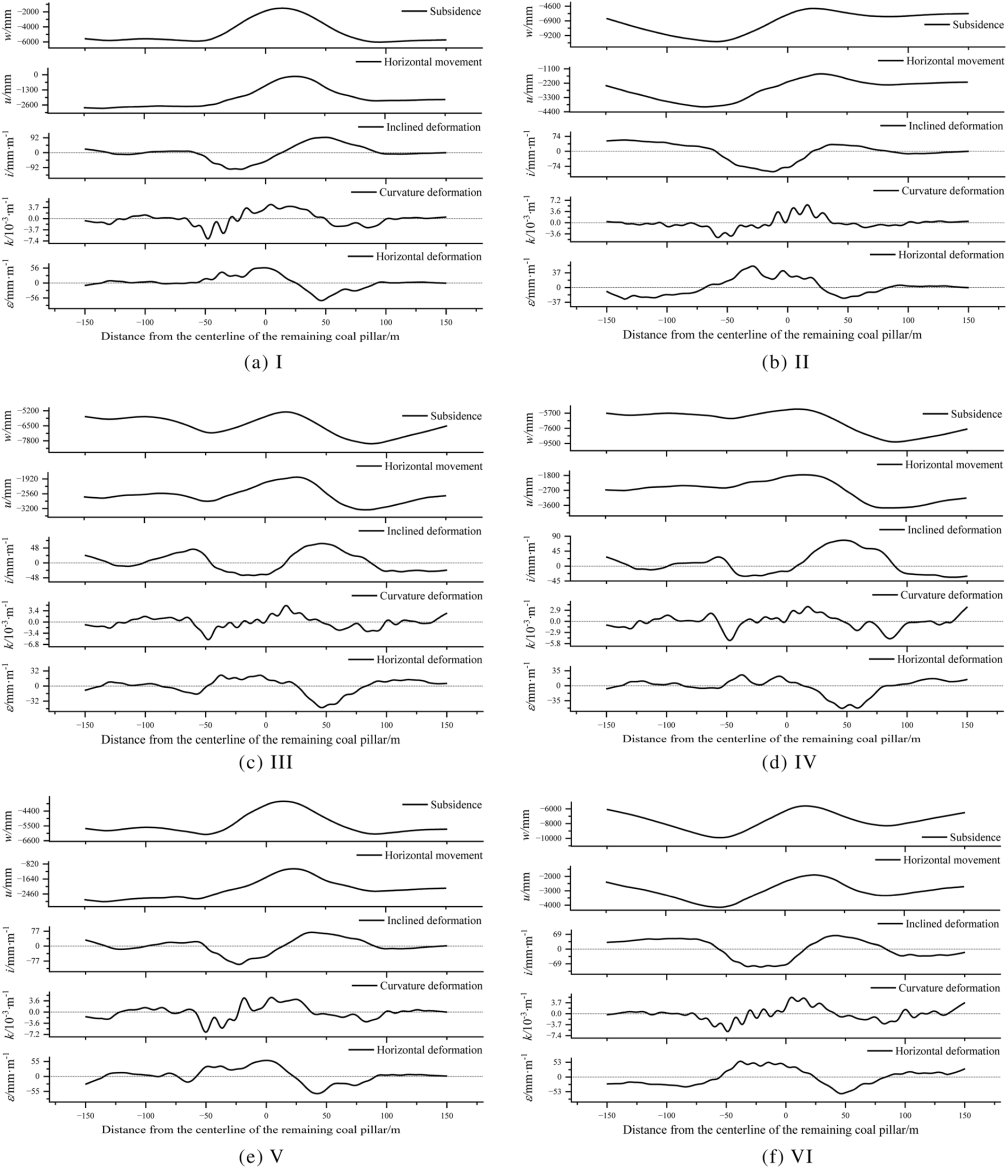
Movement and deformation curve of the No.1 coal seam.

The difference in the movement deformation curve of the No.1 coal seam is mainly reflected in the location affected by the remaining coal pillars. Obtain the movement data of the No.1 coal seam in 6 sets of experiments and calculate their movement and deformation curves, as shown in Fig. 10.

### The intensity of movement and deformation of the No.1 coal seam

**Figure 11:**
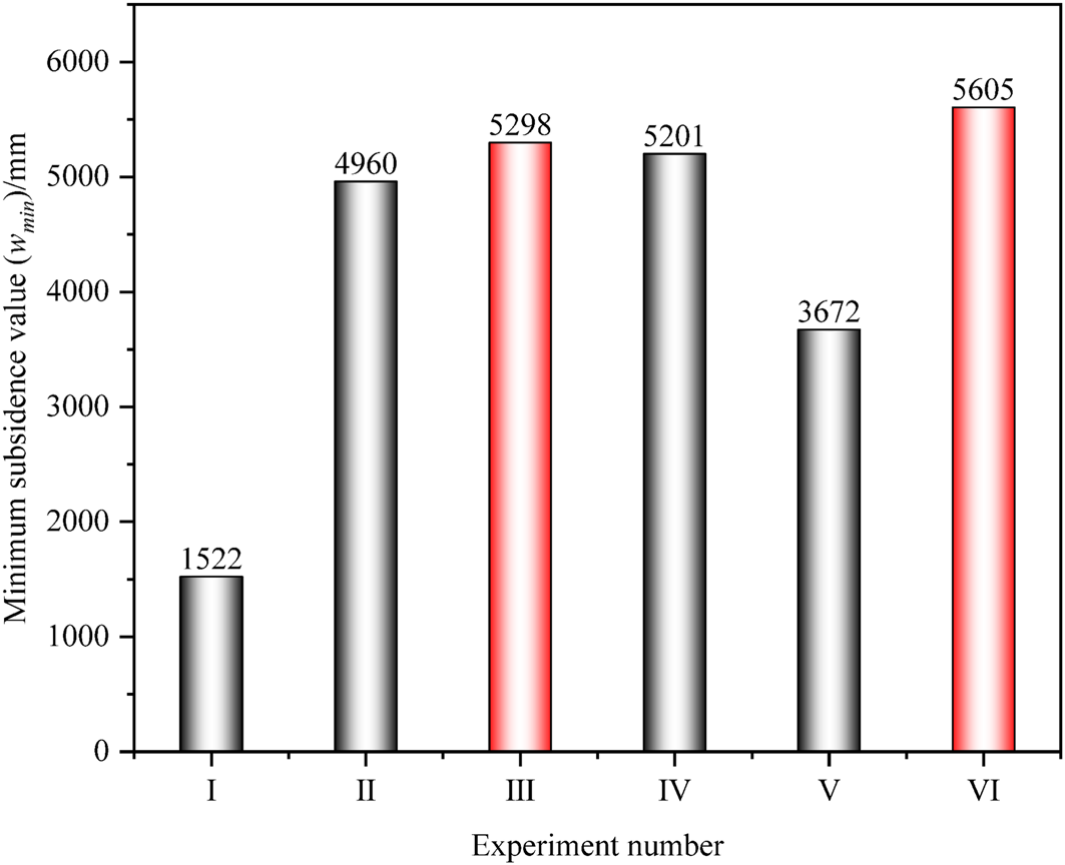
Statistics of the minimum subsidence value of the No.1 coal seam.

According to Fig. 10, count the absolute value of the movement of the No.1 coal seam within the influence range of the remaining coal pillars. Mining No.6 and 7 coal seams will inevitably lead to further movement of the No.1 coal seam. The larger the mining range, the greater the degree of overlying rock movement, which is consistent with the general law of overlying rock movement after multiple mining. From the subsidence degree of the No.1 coal seam. In Experiment VI, due to its largest mining range, it caused the strongest damage to the overlying rock, and the subsidence of the No.1 coal seam was the most sufficient, with a minimum subsidence value of 5605 mm. Next is Experiment III, where the minimum subsidence value of the No.1 coal seam is 5298 mm. In Experiment V, due to its relatively small mining range and weak damage to the overlying rock, the subsidence value of the No.1 coal seam was only 3672 mm, which is far inferior to the other four groups of experiments. This indicates that the mining of No.6 and 7 coal seams did not achieve sufficient subsidence of the No.1 coal seam, consistent with the previous analysis, as shown in Fig. 11.

**Figure 12:**
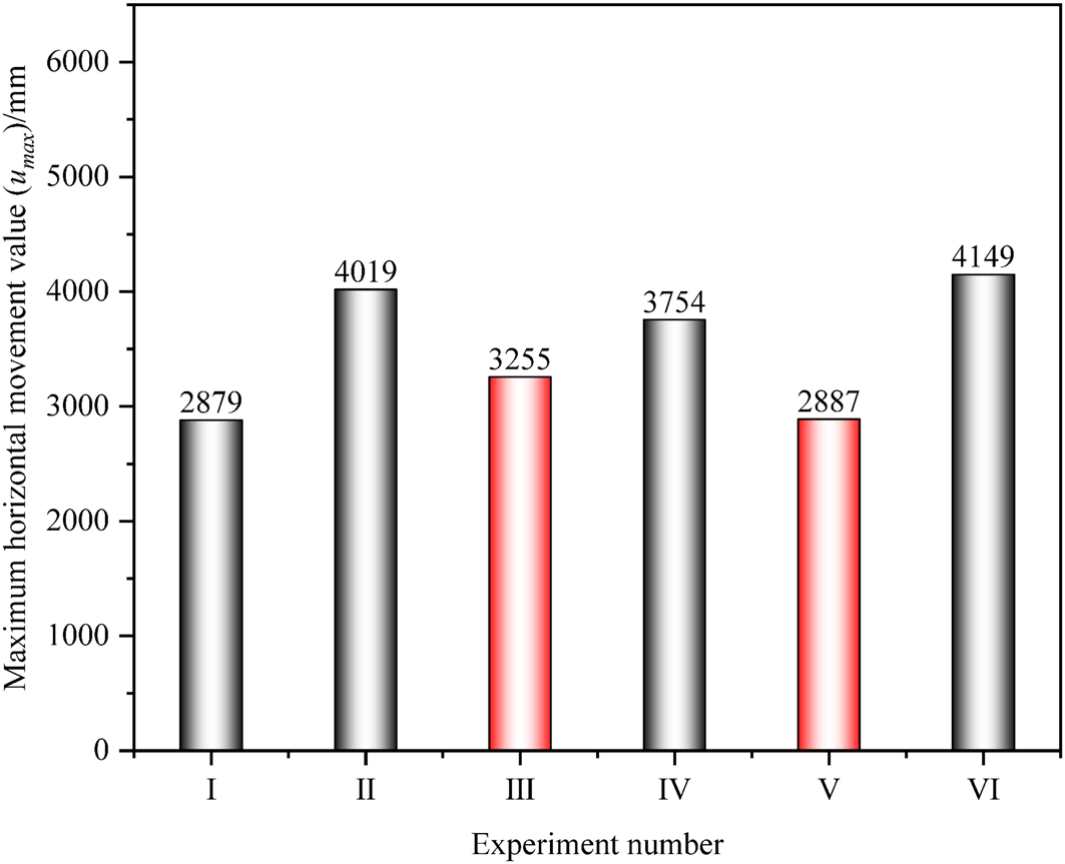
Statistics of the maximum horizontal movement value of the No.1 coal seam.

From the horizontal movement degree of the No.1 coal seam, the horizontal movement value of Experiment V is the smallest, at 2887 mm. This is due to its relatively small mining range and weak damage to the overlying rock. Next is Experiment III, which is 3255 mm. Therefore, from the perspective of the intensity of the movement of the No.1 coal seam, the layout of the working face in Experiment III can reduce the intensity of its horizontal movement while sufficient subsidence of the No.1 coal seam, as shown in Fig. 12.

**Figure 13:**
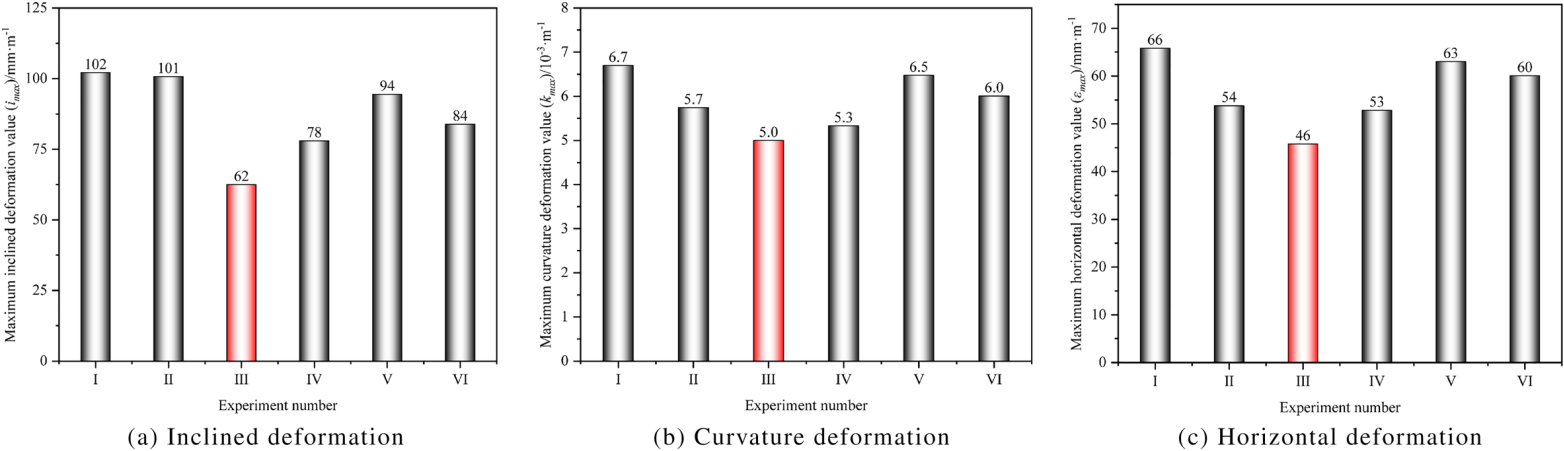
Statistics of maximum deformation values of the No.1 coal seam.

After the mining the No.6 and 7 coal seams, the intensity of deformation in the No.1 coal seam decreased. Among the six experiments, the deformation values of the No.1 coal seam in Experiment III were the smallest, with values of 62 mm m^−1^, 5.0 × 10^−3^ m, ^1^ and 46 mm·m, ^1^, respectively, as shown in Fig. 13. From the intensity of deformation, the working face parameters in Experiment III are the optimal solution. It is worth noting that when reasonable layout parameters are adopted for the working faces of the No.6 and 7 coal seams, although the further movement of the No.1 coal seam cannot be avoided, it can effectively offset the large deformation problem of the No.1 coal seam caused by the influence of remaining coal pillars. Compared to Experiment I, the reduction in deformation values of the No.1 coal seam in Experiment III was 39.2%, 25.4%, and 30.3%, respectively.

### Uniformity of movement and deformation of the No.1 coal seam

Evaluate the uniformity of the movement and deformation of the No.1 coal seam in 6 sets of experiments using the coefficient of variation^29,30^. In statistics, the coefficient of variation is a normalized measure of the degree of dispersion of a probability distribution. The smaller the value of the coefficient of variation, the smaller the degree of dispersion of the numerical value, and the more uniform it is. It is calculated using the formula (8).

8 $$ C_{v} = \frac{{\sqrt {n\sum\nolimits_{i = 1}^{n} {\left( {x_{i} - \mu } \right)^{2} } } }}{{\sum\nolimits_{i = 1}^{n} {x_{i} } }} \times 100\% $$

In the formula, *n* is the number of measuring points; *x*_i_ is the observed value of the measuring point; *μ* is the mean of all observed measurements at the measuring points.

**Figure 14:**
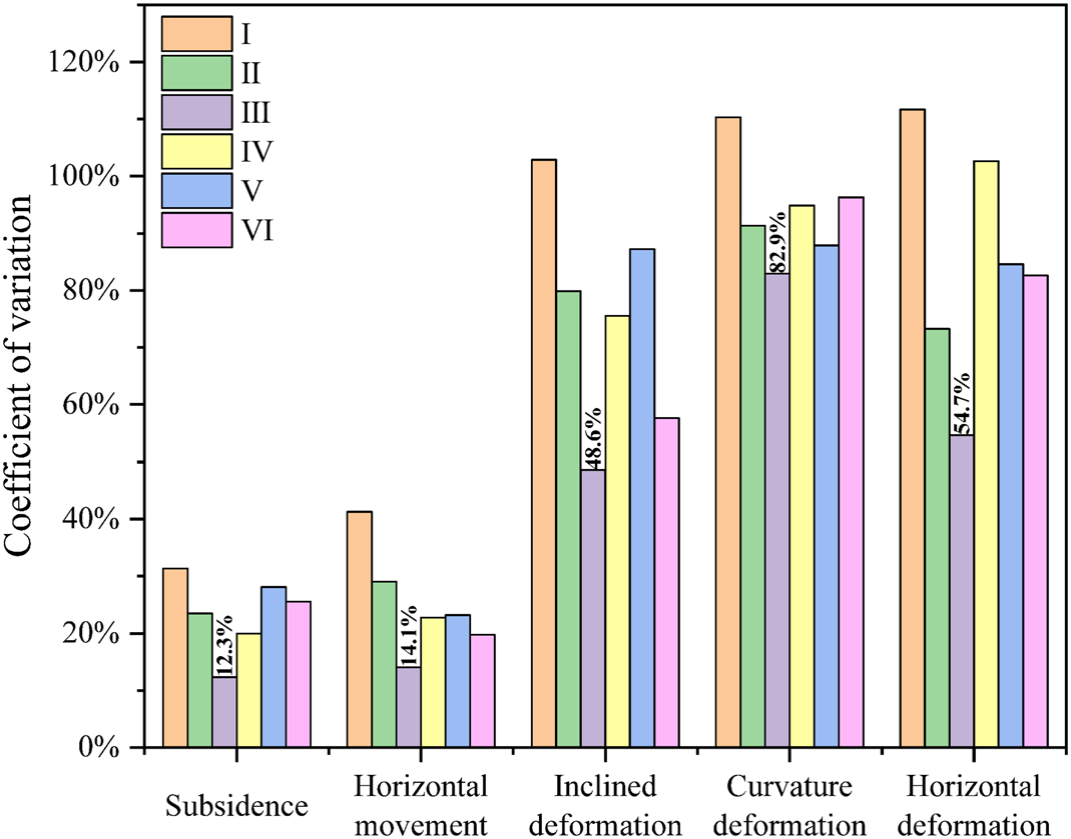
Coefficient of variation coefficient of movement deformation of the No.1 coal seam.

According to Eq. (3), calculate the coefficient of variation of the movement and deformation curves of the No.1 coal seam in Fig. 10, as shown in Fig. 14. After mining the No.6 and 7 coal seams, the overall uniformity of movement and deformation of the No.1 coal seam is smaller than that of only mining the No.2 coal seam (Experiment I); this further proves that multiple mining operations can enhance overlying rock movement and deformation uniformity. To a certain extent, it can become a favorable condition for upward mining of the No.1 coal seam. When the No.6 and 7 coal seams are within the reasonable range of the working face layout obtained in this article (Experiment III), the movement and deformation indicators of the No.1 coal seam all reach the most uniform degree of the geological mining conditions. Compared to Experiment I, the decrease in coefficient of variation in Experiment III can reach 60.7%, 65.9%, 52.7%, 9.2%, and 51.0%, respectively.

In summary, regardless of the intensity and uniformity of the movement and deformation of the No.1 coal seam, when the working faces of the No.6 and 7 coal seams are within the reasonable layout range proposed in this article, the goal of reducing the damage to the No.1 coal seam can be achieved. Multiple mining disturbances can also become favorable conditions for upward mining, verifying the rationality of the calculation method.

## Conclusion

1. Compared to the compacted area in the middle of the goaf, due to the influence of the remaining coal pillars and the hinged structures on both sides, the coal seam waiting for upward mining produces significant fluctuations, which is a disadvantageous factor for upward mining. The larger the overlying rock's collapse angle, the larger its impact's scope. The essence of reducing the damage to the coal seams waiting for upward mining is to adjust the relative positions and parameters manually between the working faces of the early mining coal seams to further subside the coal seams waiting for upward mining in the area affected by the remaining coal pillars and hinge structures and reduce the fluctuation and unevenness of the coal seams waiting for upward mining. Drawing on the concept of harmonic extraction and based on the characteristics of overlying rock movement during coal seam mining, a calculation method for the layout parameters of coal seam working face early mining is given.
2. Based on the occurrence conditions of coal seams in the fifth panel of Zaoquan Coal Mine, similar model tests were conducted to obtain the basic parameters of overlying rock movement and determine the reasonable layout parameters of the working face. The distance (*l*_1_) between the upper boundary of the No.6 and 7 coal seams working face and the centerline of the remaining coal pillar in the No.2 coal seam should be between [69.8 m, 129.9 m], the lower boundary (*l*_2_) should be between [81.0 m, 126.1 m], and the length of the working face (*l*_1_ + *l*_2_) should be between [150.8 m, 256.0 m].
3. 6 sets of numerical simulation experiments were conducted based on the occurrence conditions of coal seams in the fifth panel of Zaoquan Coal Mine. When the working face is within a reasonable layout range, it can effectively offset the large deformation problem of the No.1 coal seam caused by the influence of the remaining coal pillars. Its movement and deformation indicators reach the most uniform degree of the geological mining conditions, reducing the damage to the No.1 coal seam and making multiple mining operations favorable for upward mining. At the same time, the correctness of the calculation method was verified.

## Discussion

This article draws inspiration from the concept of “Harmonic Extraction” and takes the occurrence conditions of coal seams in the fifth panel of Zaoquan Coal Mine as the background. From the perspective of overlying rock movement, it mainly studies the problem of reducing the damage of coal seams waiting for upward mining when multiple coal seams are being mined. An analysis was conducted on the reasonable layout parameters of working faces of the early mining coal seams, and the key lies in determining the movement parameters of the overlying rock after coal seam mining. In essence, the thickness of the early mining coal seams is also a significant factor in reducing the damage to the coal seam waiting for upward mining. Meanwhile, when the spacing between coal seams is small, the impact of remaining coal pillars on overlying rock failure must be addressed, and further research is needed.

## Data Availability

The datasets generated during the current study are not publicly available. Because these data are part of our research project, which is currently under way. We must wait until this research project is completed before we can make all the data public. But the data in this paper are available from the corresponding author on reasonable request.
